# Core-Sheath Pt-CeO_2_/Mesoporous SiO_2_ Electrospun Nanofibers as Catalysts for the Reverse Water Gas Shift Reaction

**DOI:** 10.3390/nano13030485

**Published:** 2023-01-25

**Authors:** Aidin Nejadsalim, Najmeh Bashiri, Hamid Reza Godini, Rafael L. Oliveira, Asma Tufail Shah, Maged F. Bekheet, Arne Thomas, Reinhard Schomäcker, Aleksander Gurlo, Oliver Görke

**Affiliations:** 1 Chair of Advanced Ceramic Materials, Institute of Material Science and Technology, Faculty III Process Sciences, Technische Universität Berlin, Straße des 17. Juni 135, 10623 Berlin, Germany; 2 Functional Materials, Institute of Chemistry, Faculty II Mathematics and Natural Sciences, Technische Universität Berlin, Hardenbergstr. 40, 10623 Berlin, Germany; 3 Chemical Engineering/Multiphase Reaction Technology, Institute of Chemistry, Faculty II Mathematics and Natural Sciences, Technische Universität Berlin, Straße des 17. Juni 124, 10623 Berlin, Germany; 4 Inorganic Membranes and Membrane Reactors, Department of Chemical Engineering and Chemistry, Eindhoven University of Technology, P.O. Box 513, 5600 MB Eindhoven, The Netherlands; 5 Low Temperature and Structure Research Institute of the Polish Academy of Science, Okólna 2, 50-422 Wroclaw, Poland; 6 Interdisciplinary Research Centre in Biomedical Materials, COMSATS University Islamabad Lahore Campus, Defence Road, Off-Raiwand Road, Lahore 54000, Pakistan

**Keywords:** electrospinning, nanofibers, core-sheath, tandem catalyst, reverse water gas shift reaction

## Abstract

One-dimensional (1D) core-sheath nanofibers, platinum (Pt)-loaded ceria (CeO_2_) sheath on mesoporous silica (SiO_2_) core were fabricated, characterized, and used as catalysts for the reverse water gas shift reaction (RWGS). CeO_2_ nanofibers (NFs) were first prepared by electrospinning (ES), and then Pt nanoparticles were loaded on the CeO_2_ NFs using two different deposition methods: wet impregnation and solvothermal. A mesoporous SiO_2_ sheath layer was then deposited by sol-gel process. The phase composition, structural, and morphological properties of synthesized materials were investigated by scanning electron microscope (SEM), scanning transmission electron microscopy (STEM), X-ray diffraction (XRD), nitrogen adsorption/desorption method, X-ray photoelectron spectroscopy (XPS), inductively coupled plasma—optical emission spectrometry (ICP-OES) analysis, and CO_2_ temperature programmed desorption (CO_2_-TPD). The results of these characterization techniques revealed that the core-sheath NFs with a core diameter between 100 and 300 nm and a sheath thickness of about 40–100 nm with a Pt loading of around 0.5 wt.% were successfully obtained. The impregnated catalyst, Pt-CeO_2_ NF@mesoporous SiO_2_, showed the best catalytic performance with a CO_2_ conversion of 8.9% at 350 °C, as compared to the sample prepared by the Solvothermal method. More than 99% selectivity of CO was achieved for all core-sheath NF-catalysts.

## 1. Introduction

Depending on the desired structural properties, one-dimensional (1D) structures could be synthesized using various methods such as electrospinning (ES), hot-filament metal-oxide vapor deposition, sacrificial-template method, etc., [1,2,3,4]. For nanofibers (NFs) as a promising 1D structure, achieving a high surface-to-volume ratio is always desirable. Among these synthesis techniques, ES is a facile method to produce several NFs materials. Moreover, ES is a straightforward and cost-effective technique to create 1D fiber structures on the scale of nanometers to several micrometers and with various shapes, including solid, hollow, core-sheath, and hierarchical structures [5,6,7]. Such flexibility and potentials are owed to the controllable parameters of the ES process. In a typical ES process, a polymeric viscous-enough solution loaded in a needle (spinneret) is exposed to an electric field provided by a high-power supply. Then, the solution is drawn to make a jet that finally results in NFs being deposited on a collector after drying the solvent out. Electrospun NFs have been utilized in a broad range of applications, such as gas sensing [8], filtration membranes [9,10], biomedicine [11,12,13], and catalysis [14,15,16]. ES as an engineering technique has the potential to produce NFs on a large scale that is a relative advantage of this technique compared to the batch chemical synthesis methods, which produce limited amounts of the material [17,18]. Moreover, NFs prepared by ES technique can be further modified to enhance physical and chemical properties in order to achieve the desired features [9]. These can be a motivation to bridge the lab-scale production of NF to large-scale production through ES, which certainly reduces the cost of synthesis. It is practical to use the ES technique for chemistry and material fabrication to reduce the price.

In this work, systematic ES NFs with the core-sheath structure were designed and used for catalytic CO_2_ conversion, a process with significant environmental and economic implications. CO_2_ can be converted to value-added products through a tandem system, including reversed water-gas shift (RWGS) and Fischer–Tropsch (FT) reactions [19]. Designing novel multi-metallic catalysts with a controllable morphological structure will play an important role in developing a complex tandem system. Nanoparticles (NPs) as multi-metallic catalysts have been widely studied in the past few years in tandem systems. However, NF core-sheaths have been investigated in much lesser extent. In this regard, designing NF core-sheaths as a base formulation, here for RWGS, can be useful for developing multi-steps reactions, as an example, CO_2_ hydrogenation. 

Various metal oxides, such as In_2_O_3_, SrTiO_3_, CeO_2_, TiO_2_, and Al_2_O_3_ have been utilized as catalysts in RWGS [20,21,22,23,24]. Among these, CeO_2_ is the promising catalytic material, which is redox-active and shows oxygen storage capacity due to abundant oxygen vacancies [19,25,26,27]. These features and the strong metal-support interactions make CeO_2_ not only interesting as a catalyst for CO generation, but also for several other applications [19,28,29,30]. CeO_2_ is widely used as an efficient support for noble metals such as Pt. Pt-supported on CeO_2_ has received a lot of attention due to the exceptionally strong metal-support interaction [31,32]. CeO_2_ provides a better Pt dispersion than other metal oxides and Pt dispersion remains intact and stable even at high temperatures [33,34]. It is revealed that the synergistic effect between Pt and CeO_2_ could improve the catalytic properties in CO_2_ hydrogenation and RWGS [35,36,37]. However, these NPs suffer from sintering and aggregation during the catalytic process, especially in harsh conditions, which causes a loss of catalytic performance [38,39,40,41,42,43]. Embedding NPs into well-designed materials, such as core-sheath structures, can effectively minimize sintering and thus enhance the catalytic performance. Sang Hoon Joo et al. developed core-shell NPs of Pt@mSiO_2_ in which Pt was surrounded by a mesoporous SiO_2_ layer to prohibit Pt from agglomeration and improve thermal stability [42]. In another study conducted by Ji Su et al., Pt was deposited on CeO_2_ NPs and covered by a mesoporous SiO_2_ shell, and used for the conversion of ethylene to propanal via tandem hydroformylation [44]. Jones et al. studied the effect of the ceria morphology employed (nanocubes and nanorods) as supports for an iron-based catalyst for CO_2_ conversion to hydrocarbons and found that ceria nanocubes provided a high olefin-to-paraffin ratio, while a higher selectivity toward hydrocarbons was achieved using ceria nanorods [45]. Tan et al. illustrated that CeO_2_ nanotube-supported Cu-Ni shows a higher catalytic performance for CO_2_ hydrogenation to methanol compared to CeO_2_ nanoparticle-supported Cu-Ni owing to the existence of abundant oxygen vacancies and exposed (100) and (110) facets [46]. Tang et al. fabricated Pt-CeO_2_ NFs using ES technique and investigated the catalytic properties toward the water–gas shift reaction obtaining a CO conversion of 98% [47]. However, Pt particles were entrapped within CeO_2_ in electrospun Pt-CeO_2_ NFs, reducing the accessibility of the Pt active sites because of the pre-mixing of Pt and CeO_2_ solutions. To overcome this issue, Lu et al. developed CeO_2_ NFs with a hierarchical porous structure, then dispersed Pt NPs on the CeO_2_ surface using a photochemical method and obtained a uniform Pt distribution on the porous CeO_2_ [48]. However, the high porosity could decrease the mechanical stability of the NFs, which makes the formation of a homogenous sheath layer challenging. Here, to obtain a uniform sheath layer around CeO_2_, nonporous smooth CeO_2_ NFs were fabricated using the ES technique. To the best of our knowledge, electrospun NF core-sheath structures have not been investigated for RWGS so far. Moreover, two different methods were used to deposit Pt on CeO_2_ NFs, including wet impregnation and solvothermal. Deposition of pre-synthesized Pt NPs on CeO_2_ NFs using the solvothermal method have not been reported before. 

In this study, we designed a systematic multi-step synthesis method for developing the core-sheath structure of Pt-CeO_2_ NFs@mSiO_2_. Non-porous smooth CeO_2_ NFs were first produced by ES technique, then two different approaches i.e., wet impregnation and solvothermal, were applied and compared for deposition of Pt NPs, and at the last step, a mesoporous SiO_2_ was homogeneously formed around the core using the sol-gel process. The obtained catalysts were tested in the RWGS. A comprehensive characterization of the materials was further carried out to investigate the Pt distribution on NFs, core-sheath morphology, and the chemical properties of metals. 

## 2. Materials and Methods

### 2.1. Materials

Polyvinylpyrrolidone (PVP, M = 1,300,000 gmol^−1^ and 29,000 gmol^−1^), ethanol (absolute, 99.5%,), and N,N-dimethylformamide (DMF, 99.5%) were purchased from Sigma-Aldrich. Cerium nitrate hexahydrate (Ce(NO_3_)_3_·6H_2_O, >98.5%) was supplied from Merck. Commercial Cerium(IV) oxide (labeled as Com-CeO_2_) was purchased from Sigma-Aldrich. Tetradecyltrimethylammonium bromide (TTAB, 98%), and tetraethyl orthosilicate (TEOS, 98%) were obtained from Sigma-Aldrich. Cetyltrimethylammonium bromide (CTAB, 99%) and ethylene glycol (EG, 99.5%) were provided by Carl Roth. Ammonia solution 25% was purchased from Chemsolute. Tetraammineplatinum (II) nitrate ([Pt(NH_3_)_4_](NO_3_)_2_) and potassium tetrachloroplatinate(II) (K_2_PtCl_4_) were obtained from Merck. Deionized (DI) water was used in all experiments, and all materials were used without further purification.

### 2.2. Fabrication of CeO_2_ NFs

#### 2.2.1. Preparation of Spinnable Solution

In a typical synthesis, 1.4 mmol of Ce(NO_3_)_3_·6H_2_O and 1.4 mmol of PVP (M_w_ = 1,300,000 gmol^−1^) were separately dissolved in 2 and 3 mL of DMF, respectively. The polymer solution was stirred at 50 °C to facilitate PVP dissolution. After 2 h, Ce(NO_3_)_3_·6H_2_O solution was added dropwise into the polymeric solution under vigorous stirring. A yellowish spinnable solution was obtained after stirring overnight and was used for electrospinning.

#### 2.2.2. Axial Electrospinning of CeO_2_

Electrospun CeO_2_ NFs were prepared using an electrospinning apparatus, Yflow^®^ 2.2.D-300. The axial electrospinning equipment consists of a high-voltage supply, a peristaltic pump, a syringe with a 21-gauge silver-coated needle, and a ground collector. The spinnable solution was loaded into the syringe and pumped to the needle at a constant flow rate. NFs were obtained by adjusting the operating parameters with a voltage of 15 kV, a needle-to-collector distance of 15 cm, and a flow rate of 0.5 mL/h. The as-spun NFs were peeled off from the collector and dried at 80 °C in an oven for 24 h. Then the dried NFs were calcined at 600 °C for 2 h with a heating rate of 1 °C/min in air to remove PVP and obtain CeO_2_ NFs. The commercial CeO_2_ powder was used for comparison and was labeled as CeO_2_-Com.

### 2.3. Synthesis of CeO_2_NF@SiO_2_ Core-Sheath Structure

#### 2.3.1. Preparation of Electrospun CeO_2_ NFs for Sol-Gel Synthesis

In a typical synthesis, 20 mg of electrospun CeO_2_ NFs mat were ultrasonicated for 10 min to separate the nonwoven NFs to access all NFs surfaces in all dimensions. The separated powder-like CeO_2_ NFs were further used in sol-gel synthesis to obtain the core-sheath structure. 

#### 2.3.2. Sol-Gel Synthesis of CeO_2_ NF@SiO_2_ (CeSi)

A sol-gel method was implemented to synthesize the core-sheath structure, according to the previous work [49]. About 20 mg of ultrasonicated CeO_2_ NFs were dispersed in 45 mL of DI water. Then, a solution of 0.62 mmol of CTAB in 30 mL ethanol was added to the CeO_2_ NFs aqueous solution. A total of 0.2 mL of ammonia solution was added dropwise to the above solution. Subsequently, 100 µL of diluted TEOS in ethanol (1 vol%) was slowly added into the solution, followed by stirring at room temperature for 6 h. A centrifugation was performed at 4000 rpm for 5 min to collect the as-synthesized core-sheaths. The solid was calcined at 360 °C for 2 h to obtain CeO_2_ NF@SiO_2_ (labeled as CeSi)_._

### 2.4. Fabrication of Pt-CeO_2_ NF@SiO_2_

#### 2.4.1. Wet Impregnation of Pt on Electrospun CeO_2_ NF 

Pt NPs were loaded on the ultrasonicated CeO_2_ NFs using the incipient wetness impregnation technique. Theoretical loading of Pt (10 wt.% and 7 wt.%) on CeO_2_ NFs was done as follows: 0.0058 mmol (2.24 mg) of [Pt(NH_3_)_4_](NO_3_)_2_ was dissolved in 500 µL of DI water. Then this solution was added dropwise into 15 mg of CeO_2_ NFs. Subsequently, wetted CeO_2_ NFs were dried using a rotary evaporator at 45 °C for 45 min at 176 mbar. In order to obtain Pt-impregnated CeO_2_ (labeled as IM-PtCe), dried [Pt(NH_3_)_4_](NO_3_)_2_/CeO_2_ NFs were kept at 150 °C overnight to stabilize Pt on CeO_2_ NFs.

#### 2.4.2. Preparation of Pre-Synthesized Pt NPs

Preparation of Pt NPs was carried out as follows. Briefly, 21.8 mg PVP (M_w_ = 29,000 gmol^−1^) and 36.8 mg TTAB were dissolved in 10 mL EG and transferred to an argon-protected three-necked flask equipped with a condenser in Argon protection. A total of 21.9 mmol (9.1 mg) K_2_PtCl_4_ was dissolved in 4 mL EG using sonication for 20 min. Then, the Pt solution was injected into the flask and stirred for 15 min with a stirring rate of 250 rpm at room temperature. Afterwards, the reaction temperature was increased to 175 °C, kept for 30 min, and then cooled to room temperature naturally. The Pt NPs dispersed in EG were used for the solvothermal method.

#### 2.4.3. Loading of Pre-Synthesized Pt on CeO_2_ NF by Solvothermal Method

To prepare the solution, a certain amount of CeO_2_ NFs was ultrasonically dispersed in 20 mL of ethanol. Then, the pre-synthesized Pt NPs dispersed in EG solution were added dropwise into the CeO_2_ NF/ethanol suspension to prepare a mixture solution of Pt/CeO_2_ NF/ethanol. The mixture was transferred into a Teflon-lined stainless steel container and placed in an oven at 140 °C for 6 h. Afterwards, the obtained solution was centrifuged at 6000 rpm for 15 min to separate the Pt-loaded CeO_2_ NFs and then, dried overnight at 150 °C. This sample was labeled as ST-PtCe and was used for further synthesis. 

#### 2.4.4. Sol-Gel Synthesis of Pt-CeO_2_@SiO_2_

**Figure 1:**
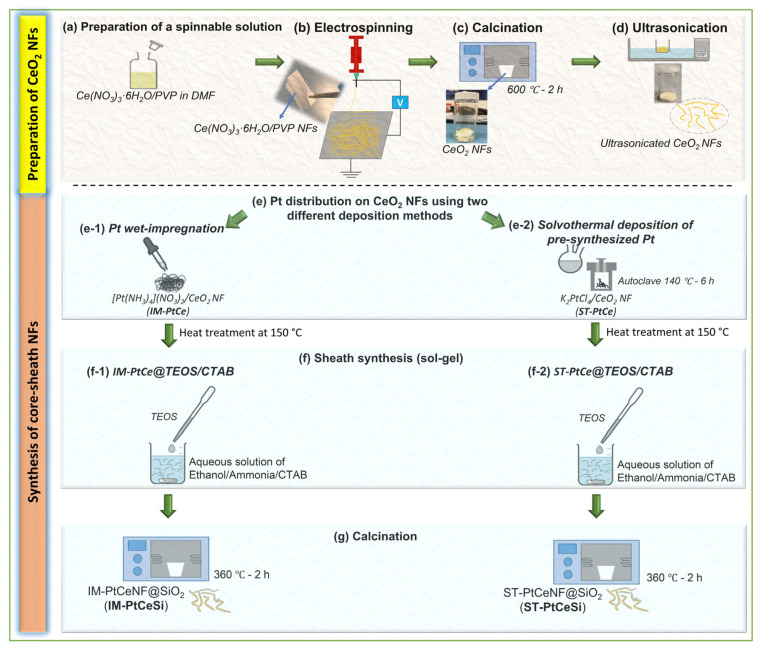
Schematic of the fabrication procedure for core-sheath NFs: (**a**) Preparation of spinnable solution of Ce(NO_3_)_3_·6H_2_O/PVP/DMF, (**b**) electrospinning of Ce(NO_3_)_3_·6H_2_O/PVP NFs, (**c**) calcination of Ce(NO_3_)_3_·6H_2_O/PVP NFs to remove PVP and obtain CeO_2_ NFs, (**d**) ultrasonication of CeO_2_ NFs, (**e**) Pt nanoparticles were deposited on CeO_2_ NFs by (**e-1**) wet impregnation using [Pt(NH_3_)_4_](NO_3_)_2_, and (**e-2**) solvothermal using pre-synthesized Pt from K_2_PtCl_4_ following by a heat treatment for all deposited NFs samples, (**f**) core-sheath synthesis using sol-gel method of (**f-1**) IM-PtCe@TEOS/CTAB, and (**f-2**) ST-PtCe@TEOS/CTAB, and (**g**) calcination of core-sheath NFs to obtain IM-PtCeSi and ST-PtCeSi catalysts.

The same sol-gel procedure (Section 2.3.2) was used to prepare the core-sheath of Pt-CeO_2_@SiO_2_. In a typical synthesis, 20 mg of Pt-CeO_2_ NFs were dispersed in 45 mL of DI water. Then as a separate solution, 225 mg of CTAB was dissolved in 30 mL of ethanol. The CTAB/Ethanol was subsequently added to Pt-CeO_2_ suspension and stirred for some minutes. About 0.2 mL of ammonia solution was added to the above solution to control the pH between 9 and 11. Subsequently, 100 µL of TEOS (diluted with ethanol) was slowly added to this solution under stirring. The core-sheath of Pt-CeO_2_@SiO_2_ was then separated by centrifuging at 4000 rpm for 5 min and dried overnight at 80 °C. Finally, the solid products were kept at 360 °C for 2 h to remove the CTAB template and to get solid oxides. Figure 1a–g schematically shows the fabrication and synthesis procedures performed in this study. All used samples described in the above sections are shown in Table 1.

### 2.5. Characterization

The crystalline phase composition of CeO_2_ NFs and core-sheath NFs after synthesis and calcination was assessed by X-ray diffraction (XRD, Bruker D8 Advance, Germany) in the reflection mode using Co K_α_ radiation (λ = 1.789 Å) in the 2θ range of 10–90° with the step size and time of 0.019° and 192 s, respectively. The indexing of crystalline phases was performed based on powder diffraction data distributed from the International Centre for Diffraction Data (ICDD^®^) [50,51]. Rietveld refinement was implemented using FullProf Suit software [52]. The refinement of all samples was performed by the profile function 7. The resolution of the instrument was provided from the structure refinement of LaB_6_ as standard. The parameters corresponding to the refinement consisted of the scale factor, zero-point of the detector, background parameters, lattice parameters, isotropic atomic displacement parameters (B_iso_), asymmetric parameters, and the fractions of side phases. 

The identification of functional groups was implemented by Fourier-transform infrared spectroscopy (FTIR) in Vertex 70 (Bruker, Germany) in the wavenumber range of 400–4000 cm^−1^. The surface composition of the materials, as well as the chemical state of the corresponding elements, was analyzed by X-ray photoelectron spectroscopy (XPS). The XPS measurement was implemented with a source gun type of Al K_α_, a spot size of 400 µm, an energy step size of 0.1 eV, and energy steps of 601 (Thermo Fischer Scientific, Waltham, MA, USA). The fitting of curves was performed using Origin 2018, and the deconvolution of the curves was performed by adjusting a shared full width at half maximum (FWHM) in a Gaussian function. All XPS spectra were corrected based on C1s binding energy of 284.8 eV. 

The specific surface area, pore size, and pore volume of NFs were investigated using N_2_ adsorption–desorption at a cryogenic temperature of 77K by QuadraSorb SI device (Quantachrome Instruments, Boynton Beach, FL, USA). The NFs were outgassed for 12 h at the temperature of 150 °C. Brunauer–Emmett–Teller (BET) theory was employed to assess the surface area of NFs. The QuadraWin software (Quantachrome Instruments, USA) was used to explore the BET data. 

The microstructure of NFs and core-sheath products were investigated by scanning electron microscopy (SEM, LEOGEMINI 1530, Zeiss, Jena, Germany). The elemental analysis was performed using energy-dispersive X-ray spectroscopy (EDS). The samples were prepared by scattering a layer of carbon to inhibit the charging during characterization. 

Morphology and fine microstructure of core-sheath structures were investigated by transmission electron microscopy (TEM), using a 200 kV LaB6 TECNAI from FEI company, operated at 200 kV and a high-resolution scanning electron microscopy (STEM), using a 300 kV cold FEG and probe-corrected JEM-ARM300F2 from JEOL Ltd., Freising, Germany, operated at 300 kV. Samples were prepared by dispersing a certain amount of electrospun and synthesized solids in ethanol using ultrasonication. Mapping analysis was implemented to evaluate the distribution of the elements in core-sheath NFs. The microscope was operated at 300 kV, equipped with a dual SDD EDX System (JEOL Ltd.) with a detection area of 2 × 158 mm^2^ and an energy resolution of 134 eV. STEM Images were acquired with a camera length of 8 cm, which corresponds to a HAADF detection angle of 68–280 mrad. 

The amount of Pt loading on CeO_2_ NFs was determined using inductively coupled plasma measurement by a Horiba Scientific ICP Ultima2 (Horiba, Kyoto, Japan).

### 2.6. Catalytic Activity Test

Reverse water gas shift (RWGS, Equation (1)) is one of the common reactions in the industry in which CO_2_ reacts with hydrogen (H_2_) to produce carbon monoxide (CO) and water (H_2_O). Due to its endothermic nature, the RWGS is favored at high temperatures [19,53]. Low-temperature CO_2_ and CO methanation (Equations (2) and (3), respectively) are side reactions of RWGS.

(1) $${\mathrm{CO}}_{2}+{\;H}_{2}\;↔\mathrm{CO}+{\;H}_{2}O\;\Delta H^{{}^{\circ}}=42.1\;\mathrm{kJ}\cdot {\mathrm{mol}}^{-1}$$

(2) $${\mathrm{CO}}_{2}+4H_{2}↔{\;\mathrm{CH}}_{4}+2H_{2}O\;\;\Delta H^{{}^{\circ}}=-165\;\mathrm{kJ}\cdot {\mathrm{mol}}^{-1}$$

(3) $$\mathrm{CO}+3H_{2}\;↔{\;\mathrm{CH}}_{4}+{\;H}_{2}O\;\Delta H^{{}^{\circ}}=-206.1\;\mathrm{kJ}\cdot {\mathrm{mol}}^{-1}$$

In this study, RWGS was carried out in a stainless-steel fixed-bed tubular column reactor (inner diameter = 4 mm and length = 70 cm). A 50 mg amount of catalyst was first diluted in 450 mg of SiC with the mesh sieve of 100–200 µm, then loaded into the reactor. The bottom of the column was packed with a layer of pure SiC (400–500 µm) and quartz wool on which the diluted catalyst was placed. The bed temperature of the reactor was measured by an installed thermocouple inside the center of the column. Before operating the catalytic activity test, the catalyst was in situ reduced at 350 °C for 2 h in the flow rates of H_2_ (40 mL/min) and N_2_ (30 mL/min) at atmospheric pressure. Next, the reactor was cooled down to the reaction temperature, and pressure was increased to 6.2 bar. The catalyst was tested under the gas flows of H_2_ (30 mL/min), N_2_ (15 mL/min), and CO_2_ (10 mL/min) with the molar ratio of H_2_:N_2_:CO_2_ (3:1.5:1) and a gas hour space velocity (GHSV) of 66,000 mL gcat^−1^ h^−1^. The operating pressure was set to 6.2 bar in all reaction temperatures. The catalyst performance was measured at three different temperatures of 250, 300, and 350 °C. The reaction was set by heating the reactor to the desired temperature at a rate of 10 °C/min. The concentrations of gas products were analyzed online by a gas chromatography instrument (Schimatzu 7890A) equipped with a thermal conductivity detector (TCD) and a flame ionized detector (FID). CO and CH_4_ were the main products of the process. The CO_2_ conversion ($X_{{\mathrm{CO}}_{2}}$) and selectivity of CO ($S_{\mathrm{CO}}$) and CH_4_ ($S_{{\mathrm{CH}}_{4}}$) were calculated through the following Equations (4)–(6):

(4) $$X_{{\mathrm{CO}}_{2}}=\frac{{\mathrm{CO}}_{2\;\left(\mathrm{in}\right)}-{\mathrm{CO}}_{2\;\left(\mathrm{out}\right)}}{{\mathrm{CO}}_{2\;\left(\mathrm{in}\right)}}\times 100\%$$

(5) $$S_{\mathrm{CO}}=\frac{{\mathrm{CO}}_{\left(\mathrm{out}\right)}}{{\mathrm{CO}}_{\left(\mathrm{out}\right)}+{\mathrm{CH}}_{4}{}_{\left(\mathrm{out}\right)}}\times 100\%$$

(6) $$S_{{\mathrm{CH}}_{4}}=\frac{{\mathrm{CH}}_{4\;\left(\mathrm{out}\right)}}{{\mathrm{CO}}_{\left(\mathrm{out}\right)}+{\mathrm{CH}}_{4\;\left(\mathrm{out}\right)}}\times 100$$

where (in) and (out) are denoted for the mole of reactant and effluent corresponding gases respectively. 

## 3. Results and Discussion

**Figure 2:**
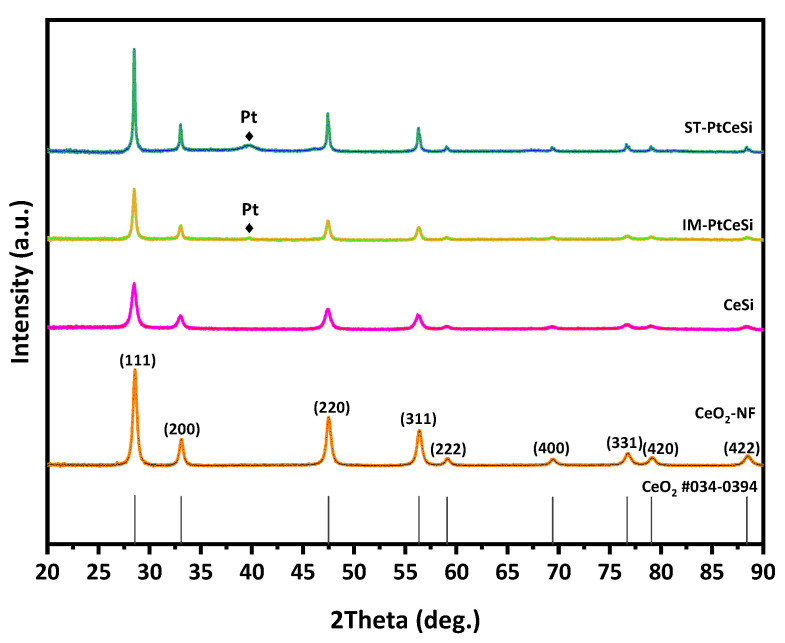
XRD patterns of CeO_2_-NFs, CeSi, IM-PtCeSi, and ST-PtCeSi samples.

CeO_2_ NFs were fabricated using ES technique, followed by a calcination step at 600 °C. The NFs were further used for synthesizing the core-sheath structure of CeO_2_@SiO_2_ via a sol-gel route. Figure 2 shows XRD patterns of all samples, including electrospun CeO_2_ NFs, CeSi core-sheath, ST-PtCeSi, and IM-PtCeSi NFs. The measured pattern for CeO_2_ NFs can be attributed to a pure CeO_2_ crystal structure based on JCPDS 34-0394 representing the fluorite cubic structure. The reflections at 28.5°, 33.14°, 47.5°, 56.5°, 59°, 69.5°, 77°, 79°, and 88.5° are associated with (111), (200), (220), (331), (222), (400), (331), (420), and (422) crystal planes of CeO_2_, respectively [48]. After synthesizing a silica layer around the electrospun CeO_2_ NFs, the intensity of XRD reflections is significantly reduced, and the diffuse scattering at 20–25° increased without the appearance of a new XRD reflection, suggesting the amorphous structure of the SiO_2_ overlayer. Similar changes were also observed in the XRD patterns of IM-PtCeSi and ST-PtCeSi samples loaded with Pt and coated with silica layers. Additionally, very tiny XRD reflections are observed at 39.8°, 46.2°, and 67.5° for both catalyst samples of IM-PtCeSi and ST-PtCeSi, which are attributed to Pt (111), (200), and (220) planes of metallic Pt, respectively. 

To gain further insight about lattice parameters, crystallite size, microstrain, and preferred orientation in the samples, Rietveld refinement was performed on the diffraction patterns. The preferred orientation was determined with the March–Dollase model [54], as implemented in the FULLPROF program. Figure S1 shows the observed, calculated, and difference profile for the final cycle of the structure refinement. The results of Rietveld refinement reveal that the lattice parameter of CeO_2_ does not change significantly with synthesizing a silica layer around the electrospun CeO_2_ NFs or Pt loading, suggesting that the silica and Pt are mainly added to the surface of the CeO_2_ NFs without altering the lattice CeO_2_ phase. In contrast, the crystallite size of CeO_2_ increases from 14.9 nm in CeO_2_ NFs to 21.3 nm and 36.5 nm in IM-PtCeSi and ST-PtCeSi, respectively, with the addition of silica and Pt. The March-Dollase (MD) parameter *r* along the <110> directions was found to be higher than unity in all samples, with increasing from 1.1 CeO_2_ NFs to 1.3 and 1.4 for IM-PtCeSi and ST-PtCeSi samples, respectively. This MD parameter *r* defines the crystallites’ habit distribution and is unity for an ideal random-orientation (i.e., no preferred orientation), greater than one for needle-habit crystals and less than one for platy crystals pack along the diffraction vector. Thus, the CeO_2_ crystals in the NFs are grown as needle-habit along the <110> directions in all samples. However, the values of MD parameter *r* indicate the percentage of excess crystallites with preferential orientation in comparison with randomly oriented crystallites in the NFs, which means that ST-PtCeSi sample has crystallites with the highest total preferential orientation along the <110>, followed by the IM-PtCeSi sample. Moreover, the weight fraction of nanocrystalline Pt metals (~4.4 nm) was found to be 2.5 (0.2) wt.% and 19.2 (0.5) wt.% in IM-PtCeSi and ST-PtCeSi samples, respectively. The higher amounts of Pt detected in the samples from XRD compared to the experimental value (7 wt.%) can be explained by the amount of amorphous silica excluded in the Reitveld refinement analysis. These results suggest that the Solvothermal deposition of Pt on the CeO_2_ NFs enhances the growth of CeO_2_ crystallites along <110> direction and the formation of a high amount of Pt nanocrystallites (4.4 nm). In contrast, although wet-impregnation of Pt increases the preferred orientation of CeO_2_ along the <110> direction, a slight increase in the crystallite size of CeO_2_ and a low amount of metallic Pt are observed. Since the same amount of Pt loading was used for both impregnation methods, the low weight fraction of Pt in the IM-PtCeSi suggests the presence of a high amount of metallic Pt with a very small crystallites size to be detected by XRD (<2 nm). 

The FTIR spectra of CeSi from 4000 to 430 cm^−1^ are illustrated in Figure S2. All absorption bands corresponding to Si-O and Ce-O groups can be seen in the FTIR spectra. The band at about 440 cm^−1^ corresponds to the Ce-O vibration. The two absorption bands at 1063 cm^−1^ and about 810 cm^−1^ can be ascribed as symmetric and asymmetric Si-O-Si bonding groups, respectively [55]. The small band at 1650 cm^−1^ can be related to O-H stretching bond. Likewise, a small absorption band can be observed at 3750 cm^−1,^ which is attributed to the OH vibrations of free silanol groups [56,57].

**Figure 3:**
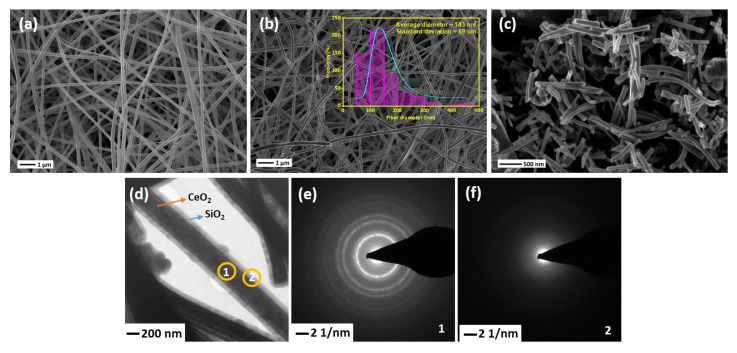
SEM images of (**a**) as-spun Ce(NO_3_)_3_·6H_2_O/PVP NFs, (**b**) CeO_2_ NFs after calcination at 600 °C, Insert shows the size distribution plots of fibers diameter, (**c**) CeO_2_ NF after 10 min ultrasonication, (**d**) TEM image of CeSi, (**e**,**f**) SAED of CeSi, core (1) and sheath (2).

SEM images of the electrospun NFs are shown in Figure 3. As-spun PVP/Ce(NO_3_)_3_·6H_2_O NFs are illustrated in Figure 3a. The average diameter of NFs before calcination is about 200–250 nm, while the diameters have been reduced to 90–100 nm after heat treatment of the NFs at 600 °C due to the removal of PVP along with other organic moieties and oxidation of Ce(NO_3_)_3_·6H_2_O into CeO_2_, as can be seen in Figure 3b. This shows that the fibrous shape of CeO_2_ NFs remains intact while removing PVP from the structure. Figure 3b shows that the surface of produced NFs is smooth with a relatively uniform average diameter of 143 nm. Prior to introducing the SiO_2_ sheath, the mat of CeO_2_ NFs was ultrasonicated for better accessibility of the entire CeO_2_ surface. Figure 3c shows that after ultrasonication, the average length of the NFs decreased to 500–1000 µm. TEM image of the CeSi core-sheath NFs confirms the successful formation of core-sheath structure (Figure 3d). A clear interface between CeO_2_ NFs as core and SiO_2_ as sheath can be identified, with a core diameter of about 340 nm and a sheath thickness of about 70 nm. Although well-designed core-sheath structures have been achieved, a few extra spherical SiO_2_ particles can be seen in some regions. These particles might have formed first separately during sol-gel synthesis and then attached on the silica sheath. To verify the crystallinity of structure, SAED analyses were performed for both core and sheath layers (Figure 3e,f). The SAED images confirm the presence of a polycrystalline CeO_2_ material with a fluorite cubic structure in the core (coded by 1) of CeO_2_ NFs and an amorphous silica sheath (coded by 2).

**Figure 4:**
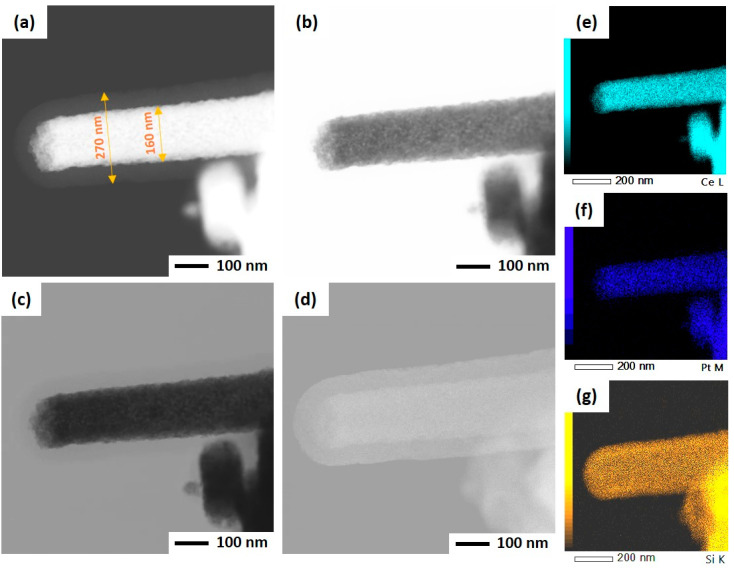
STEM of IM-PtCeSi in different imaging: (**a**) high-angle annular dark field mode, (**b**) annular bright-field mode, (**c**) bright-field mode, (**d**) secondary electron mode, (**e**) EDS elemental mapping of Ce, (**f**) EDS elemental mapping of Pt, and (**g**) EDS elemental mapping of Si.

**Figure 5:**
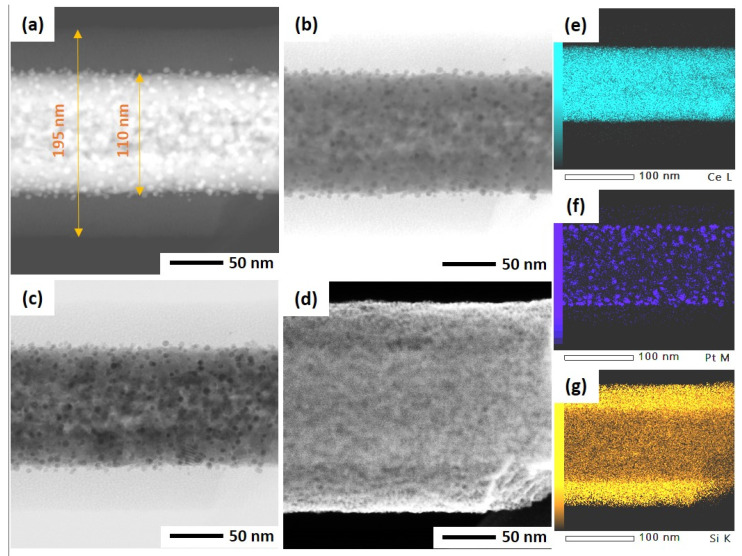
STEM of ST-PtCeSi in different imaging: (**a**) high-angle annular dark field mode, (**b**) annular bright-field mode, (**c**) bright-field mode, (**d**) secondary electron mode, (**e**) EDS elemental mapping of Ce, (**f**) EDS elemental mapping of Pt, and (**g**) EDS elemental mapping of Si.

As mentioned before, the ultrasonicated electrospun CeO_2_ NFs have been used to support Pt nanoparticles by two different methods: wet impregnation and solvothermal deposition. In the impregnation method, Pt was directly deposited on CeO_2_ NFs, while PVP and TTAB were used as capping agent and template for synthesis of Pt NPs and its deposition on CeO_2_. After the deposition of Pt, a silica sheath was grown around the Pt-CeO_2_ NFs to obtain the core-sheath structure. STEM was used to investigate the Pt distribution and core-sheath morphology. Distinguishable interfaces between core and sheath can be observed for IM-PtCeSi with the theoretical 10 wt.% of Pt loading on CeO_2_ NF. The core diameter is about 100 nm, and the sheath thickness is around 40 nm (Figure S3). Elemental mapping of Ce, Si, O, and Pt was also performed and is shown in Figure S3. As can be seen, Pt presents in some areas of the sheath layer. To improve the Pt stabilization on the CeO_2_ core, a heat treatment at 150 °C for 24 h was carried out after wet impregnation. Furthermore, the theoretical loading of Pt on CeO_2_ NF was decreased from 10 wt.% to 7 wt.% for IM-PtCeSi. As shown in Figure 4, the successful formation of the core-sheath structure and the well distribution and stabilization of Pt on CeO_2_ were obtained. It can be concluded that no migration of Pt into the sheath has taken place. The elemental mapping analysis of IM-PtCeSi confirms as well that Pt is well distributed on CeO_2_. The diameter of core-sheath NF and the sheath thickness were about 160 nm and 55 nm, respectively. Solvothermal deposition of pre-synthesized NPs on metal oxides has been utilized to increase the interaction between metal and support [58]. So, in another approach, solvothermal deposition of Pt on CeO_2_ NFs was adopted to further enhance the distribution and stabilization of Pt on the substrate. Figure S4 illustrates STEM images of ST-PtCeSi sample which also shows the migration of Pt into the SiO_2_ sheath. To improve the Pt stabilization and prevent its detachment from the surface of CeO_2_ NFs, a heat treatment at 150 °C for 24 h was applied after the solvothermal method, Figure 5 and Figure S5. A well-defined core-sheath structure is obtained with a core diameter of about 110 nm and a sheath thickness of about 42 nm. 

The particle size of Pt in ST-PtCeSi was measured to be 3.8 nm, consistence with crystallite size determined from XRD analysis (4.4 nm). The particle size of the pre-synthesized Pt NPs used in the solvothermal method is larger than that used in the wet impregnation method, Figure S5, and it agrees with XRD results. Considering Figure 4, it can be concluded that the Pt particle size in IM-PtCeSi is less than 3.8 nm. 

**Figure 6:**
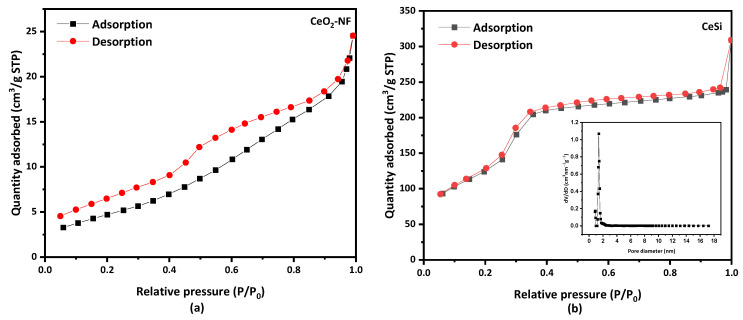
Nitrogen adsorption–desorption isotherm of (**a**) electrospun CeO_2_ NFs, and (**b**) core-sheath of CeSi, insert graph shows pore diameter of CeSi.

Figure 6 shows the nitrogen adsorption–desorption isotherms for electrospun CeO_2_ NFs with a specific surface area of 18 m^2^·g^−1^. A type IV BET isotherm is obtained for CeSi (Figure 6a), from which a surface area of 476.5 m^2^·g^−1^ can be calculated. While a type IV of BET isotherm appeared for the core-sheath sample of CeSi with a hysteresis loop, Figure 6b. The significant increase in surface area can be attributed to the presence of the porous SiO_2_ layer. The inset graph in Figure 6b shows the Barret–Joyner—Halenda (BJH) pore size distribution curve for CeSi, which gives a pore diameter of about 2.8 nm for the silica layer.

**Figure 7:**
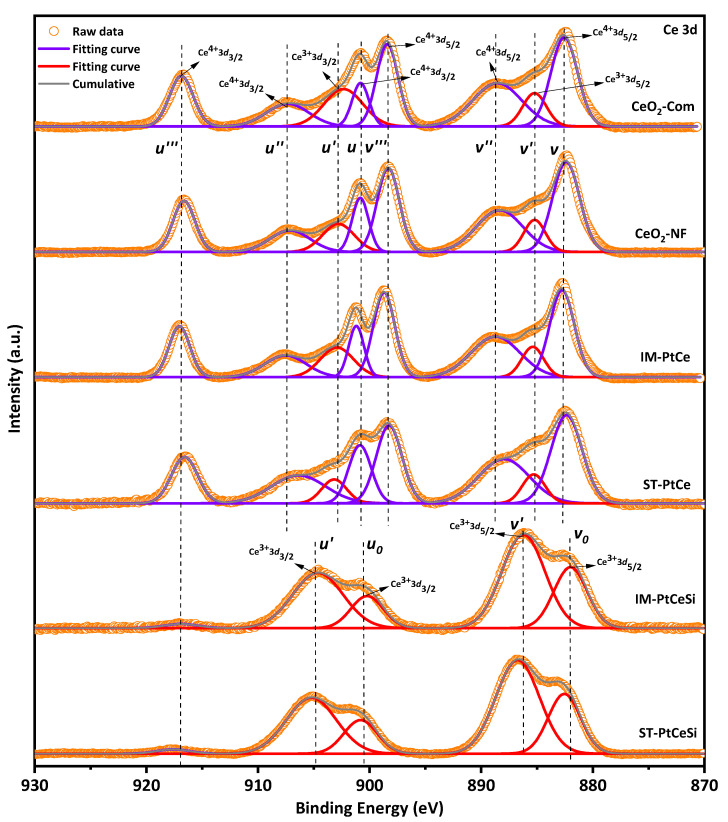
Ce 3d X-ray photoelectronic spectra for different samples.

For a better understanding of the chemical states of samples, the XPS analysis was performed and compared with CeO_2_-Com. Figure 7 and Figure S6 show the XPS spectra of Ce 3d and O 1s [59,60,61,62,63,64], respectively, for CeO_2_-Com, CeO_2_ NF, IM-PtCe, SN-PtCe, IM-PtCeSi, and ST-PtCeSi. Multiplets of u and v corresponding to the spin-orbital splitting of Ce 3d_3/2_ and 3d_5/2_ are observed in Figure 7. The spin-orbit splitting of Ce 3d is reported to be about 18.4 eV [65]. Typically, the Ce 3d spectrum displays five doublet pairs. The doublet pairs of (u_0_-v_0_), (u′-v′) ascribes to Ce(III), whereas (u-v), (u″-v″), and (u‴-v‴) are ascribed to Ce(IV) [66,67]. The doublets with corresponding oxidation states are indicated in the XPS spectrum for each sample. In the case of CeO_2_ NFs, in addition to the Ce^4+^ peaks, the peaks with the binding energies (BEs) of about 903 and 885 eV indicate the presence of Ce^3+^ species in the electrospun CeO_2_ NFs. Both oxidation states are also observed in CeO_2_-Com. 

The fractional amount of Ce^4+^ and Ce^3+^ was calculated using Equations (S1)–(S3) [66]. As shown in Figure S7, the commercial CeO_2_ powder consists of 24.4% of Ce^3+^ and 75.6% of Ce^4+^, while a Ce^3+^ concentration of 13.6% was obtained for electrospun CeO_2_-NFs. During wet impregnation of Pt on the CeO_2_-NFs, the amount of Ce^3+^ species increases to 20.4%, indicating that more oxygen vacancies have been generated. Similarly, the fraction of Ce^3+^ species in the ST-PtCe sample rises to 17.5%. The higher oxygen vacancies observed in IM-PtCe might be related to the more uniform distribution of Pt on the surface of CeO_2_ NFs compared to ST-PtCe (see Figure 4 and Figure 5). Since XPS is a surface-sensitive technique, a core-level etching XPS of the core-sheath NFs was performed to obtain precise information for Ce species. As can be seen in the spectra of IM-PtCeSi and ST-PtCeSi (Figure 7), the intensity of the peaks at 881.8 eV, 900.3 eV, 916.5 eV related to Ce^4+^ significantly decreases, whereas for Ce^3+^ increases, indicating that the oxidation state of Ce in the vicinity of SiO_2_ sheath was changed to non-stoichiometric (CeO_2−X_) [68,69,70]. The observed reduction of CeO_2_ on the surface can be likely attributed to the presence of free OH^−^ groups in the synthesis medium during the SiO_2_ sheath formation. As discussed in Rietveld refinement analysis (Figure S1), the lattice parameter of CeO_2_ was not significantly changed in all samples meaning that the reduction of Ce^4+^ to Ce^3+^ did not occur in the bulk of the materials and considering to the XPS analysis, it can be concluded that Ce^3+^ species was merely attributed to the surface.

**Figure 8:**
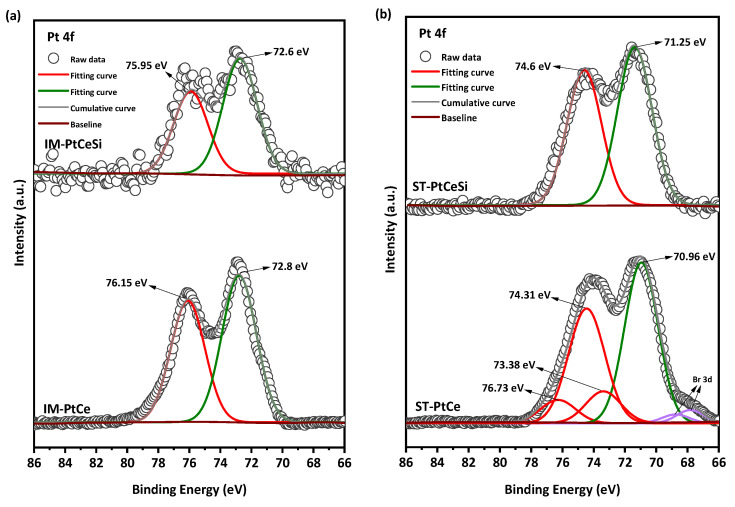
Pt 4f X-ray photoelectronic spectra of (**a**) IM-PtCe and IM-PtCeSi, and (**b**) ST-PtCe, and ST-PtCeSi.

The Pt 4f region (Figure 8) is deconvoluted into two spin-orbit split doublets of 4f_5/2_ and 4f_7/2_. All peaks in IM-PtCe are attributed to the oxidized form of Pt with the characteristic peaks at 75.95 eV (${\mathrm{Pt}}_{5/2}^{2+}$) and 72.6 eV (${\mathrm{Pt}}_{7/2}^{2+}$) [71,72,73,74,75,76]. The oxidation of Pt in IM-PtCe can be due to the heat treatment before growing the silica sheath. For the core-sheath sample of IM-PtCeSi, an etching-XPS measurement was performed to access the Pt deposited on the CeO_2_ core. The spectra looks similar and comparable to IM-PtCe. The Pt 4f spectra for ST-PtCe and ST-PtCeSi are shown in Figure 8b. In ST-PtCe, Pt exposes two doublets at the BEs of 70.96 and 74.31 eV related to the metallic form of Pt (${\mathrm{Pt}}_{7/2\;}^{0}{\mathrm{and}\;\mathrm{Pt}}_{5/2}^{0}$), and BEs of 73.38 eV and 76.73 eV associated with the oxidized forms of Pt. A tiny doublet corresponding to Br3d present in ST-PtCe spectra at the BEs of 67.74 eV and 68.78 eV [77], can be attributed to bromide ions in TTAB used for the preparation of pre-synthesized Pt and left on CeO_2_ NFs surface. After introducing the SiO_2_ sheath and heat treatment at 360 °C, the peaks at the BEs of 71.25 eV, 74.6 eV were observed in ST-PtCeSi, which can be assigned to the metallic form of Pt [78,79]. The larger particle size in ST-PtCe and ST-PtCeSi causes a peak shift to lower BE, as also has been reported in the literature [80]. The peak positions, oxidation states, and corresponding integrated areas for Ce and Pt are summarized in Tables S1 and S2.

To investigate the basicity of the materials, CO_2_-TPD was performed for IM-PtCeSi and ST-PtCeSi and presented in Figure S8. The high-temperature desorption peaks observed at 550 and 650 °C for ST-PtCeSi and IM-PtCeSi, respectively, reveal relatively strong basicity in both catalysts. The higher desorption temperature for IM-PtCeSi confirms its stronger basicity compared to ST-PtCeSi at high temperature. Moreover, the desorption peak for IM-PtCeSi is much more intense than ST-PtCeSi, revealing a higher amount of adsorption centers for CO_2_ molecules. 

**Figure 9:**
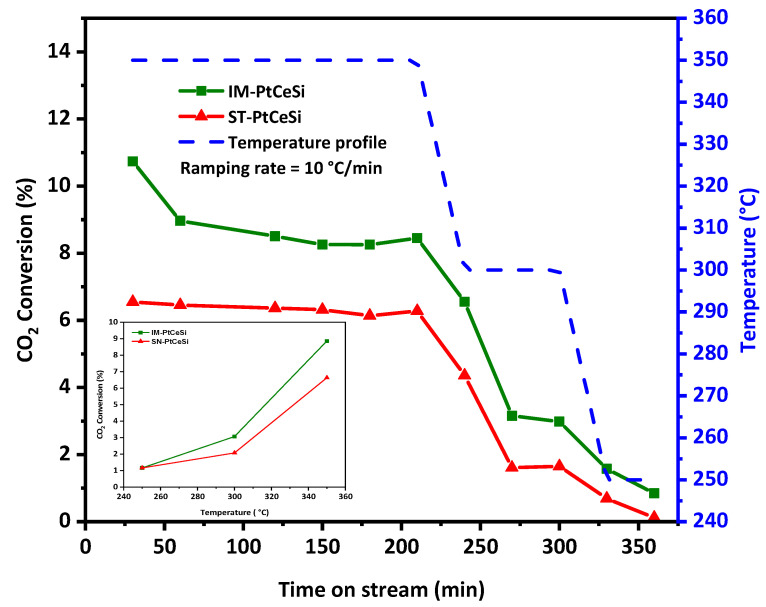
Catalytic testing of IM-PtCeSi and ST-PtCeSi, CO_2_ conversion, and temperature profile vs. time on stream for RWGS. Inset figure: CO_2_ conversion (in average values) vs. temperature.

Both core-sheath catalysts, IM-PtCeSi and ST-PtCeSi, were tested for the RWGS reaction at different temperatures, 250, 300, and 350 °C. Figure 9 shows the temperature profile and CO_2_ conversion during the reaction. The selectivity of CO and CH_4_ for both catalysts is illustrated in Figure S9. As RWGS is an endothermic reaction, the CO_2_ conversion increases with increasing temperature [81,82,83]. The highest CO_2_ conversion of 9% and 6.7% was obtained for IM-PtCeSi and ST-PtCeSi, respectively, at 350 °C. Figure S10 shows the equilibrium conversion of CO_2_ between 200 and 600 °C. The experimental CO_2_ conversions obtained in this study, are less than the equilibrium one, for example, at 350 °C a CO_2_ conversion of 30% can be achieved at the equilibrium conditions. The CO_2_ hydrogenation mechanism has been reported previously [35], in which CO_2_ can be converted through the redox mechanism and directly reacts with the oxygen vacancies in CeO_2_, producing CO. In this case, Pt mainly contributes to generate oxygen vacancies by activating H_2_, and then the oxygen vacancies migrate to CeO_2_. XPS spectra of core-sheath samples (Figure 7) confirmed the existence of oxygen vacancies in the corresponding interface of ceria core and SiO_2_ sheath. Moreover, IM-PtCe possesses higher Ce^3+^ species (Figure S7), so more oxygen vacancies are generated in this sample, therefore it can be expected that the catalytic performance of IM-PtCeSi becomes higher than ST-PtCeSi. In addition, due to the fact that Pt can spill over hydrogen to the neighboring CeO_2_ [84], smaller particle size and higher distribution of Pt can lead to more hydrogen placing in its coordination to CeO_2_, which might enhance the catalytic performance. The smaller Pt particle size and its high distribution present in IM-PtCeSi sample compared to ST-PtCeSi can provide a higher surface area for Pt particles on CeO_2_ NF that improves corresponding catalytic efficiency. With regard to CO_2_-TPD results, IM-PtCeSi shows higher CO_2_ uptake compared to St-PtCeSi (Figure S8), thus CO_2_ has more opportunity to be reduced to CO in IM-PtCeSi catalyst leading to its higher catalytic performance. Moreover, the presence of a smaller crystallite size of CeO_2_, grown along the <110> direction, in IM-PtCeSi catalyst compared to ST-PtCeSi could be another reason for higher catalytic performance. Tan et al. reported that the exposed (110) facets and the existence of abundant oxygen vacancies in CeO_2_ nanotube-supported Cu-Ni contribute to its higher catalytic performance for CO_2_ hydrogenation [46]. Table 2 summarizes the performance of different tested catalysts for RWGS with various catalysts and the Pt loadings% of 0.025 to 2%wt, showing that increasing the loading amount of Pt enhances the CO_2_ conversion. Considering Table 2, the obtained CO_2_ conversions in our study are comparable to the catalysts with the same Pt loading tested at a similar range of temperatures [23,24,36,37,85,86,87,88]. Besides the catalyst type, several parameters corresponding to reactor configuration (reactor type, reactor dimension, etc.,) and operation conditions (temperature, pressure, GHSV, feed ratio) can also affect the catalytic performance; therefore, a precise comparison study is challenging in RWGS. A conversion of 6.7% has been reported by Chen et al. for Pt-CeO_2_ nanoparticles with the Pt loading of 1 wt.% at 300 °C [36], whereas in this study, core-sheath NFs showed less conversion with 0.5 wt % Pt loading in a higher GHSV, at the same temperature. In another study performed by Zhao et al. [37], 8.5% CO_2_ conversion with more than 98% CO selectivity had been reported approximately at similar testing conditions, while the core-sheath catalysts tested in our study showed nearly the same conversion, 9%, and a higher CO selectivity of 99%. Considering this comparison, it can be assumed that the access of the reactants to the core is not completely prohibited by silica sheath and Pt active sites are accessible. Pt-CeO_2_@SiO_2_-Co spherical NPs have been studied for CO_2_ hydrogenation tandem systems to olefins. A CO_2_ conversion of 25% and more than 98% selectivity could be achieved at 350 °C over Pt-CeO_2_@SiO_2_ nanoparticles for RWGS reaction with the loading of 4.4% Pt [49]. Whereases in this study, the core-sheath NFs showed conversions of 9% and 6.8% with IM-PtCe and ST-PtCeSi, respectively, with around nine times lower Pt loading. Although the obtained conversion in this study is not very high considering the thermodynamic conversion, the synthesis approach can be optimized in future to improve the catalytic performance. The proposed catalysts can be potentially used in a tandem system as a basic structure by introducing a second active metal on the sheath layer. It should be noticed that in a tandem system such as CO_2_ hydrogenation, the obtained conversion of RWGS should be optimized considering the second active site to reach the best ratio of subsequent reactions. Based on the systematic synthesis approach developed in this article, the proposed method can be used to synthesize different catalysts for a variety of tandem application. 

In order to investigate the morphology of the tested catalysts, a TEM analysis was performed after reaction, Figure S11. As seen, the morphology of catalysts is not changed, and a clear interface between the core and sheath can be observed in both catalysts. This indicates that the core-sheath structures remained intact under the catalytic testing conditions. However, Pt aggregation has been observed for ST-PtCeSi, Figure S11d.

## 4. Conclusions

The electrospinning technique has the potential to produce NFs on a large scale, compared to the chemical syntheses in which the product might be yielded on limited scales. The NFs morphology can also be tuned using this technique so that potentially a bulk texture of nonwoven NFs can be directly utilized for further surface modifications and tested in a reactor for catalysis. The electrospun NFs can provide a higher surface area which might improve the catalytic performance. In this work, core-sheath NFs of Pt-CeO_2_ NF@mSiO_2_ were successfully fabricated using the electrospinning technique and further sol-gel synthesis. The sheath layer of SiO_2_ was grown directly on electrospun NFs without using a capping agent. Pt was deposited on CeO_2_ NFs by two different methods, including wet-impregnation and solvothermal deposition of pre-synthesized Pt. Structural and morphological studies revealed that Pt was more homogeneously dispersed on CeO_2_ NF in IM-PtCeSi compared to ST-PtCeSi. The results of Rietveld refinement of XRD data revealed that the Pt impregnation led to the formation of small weight fractions of metallic Pt nanoparticles (≤4.4 nm) without inducing a significant change in the lattice parameter of CeO_2_, indicating that the Pt is mainly added to the surface of the CeO_2_ NFs without altering the lattice CeO_2_ phase. Although the CeO_2_ crystals in the NFs were grown as needle-habit along the <110> directions in all samples, Pt impregnation increased the preferred orientation of CeO_2_ along this direction. The XPS results regarding Ce 3d showed that the oxygen vacancies were increased by reducing Ce^4+^ species to Ce^3+^ in IM-PtCe after wet impregnation of Pt on CeO_2_ NF. A uniform and intact porous SiO_2_ sheath layer were obtained in both catalyst samples. Both catalysts were then tested for the RWGS reaction. IM-PtCeSi showed better performance compared to ST-PtCeSi, with a CO_2_ conversion of 8.9% and a CO selectivity of 98.9% (at P = 6.2 bar, 350 °C, GHSV = 66,000 mLgcat^−1^h^−1^). It is also demonstrated that by increasing the operating temperature, the catalytic performance is enhanced in all catalysts. Such a porous silica layer can be potentially used as a second substrate and interlayer to design tandem bifunctional catalysts as well.

## Figures and Tables

**Table 1:** Prepared samples with or without Pt loadings by electrospinning (ES), wet and solvothermal impregnation and sol-gel methods. The table is visually represented in Figure 1.

Sample	Material/Precursor	Preparation Method(s)	Heat Treatment/ Calcination Temperature (°C)	Desired Structure
Com-CeO_2_	Commercial CeO_2_	-	-	CeO_2_ powder
Ce(NO_3_)_3_·6H_2_O/PVP NF	Ce(NO_3_)_3_·6H_2_O/PVP	ES	No	Composite polymer/metal nitrate fibers
CeO_2_ NF	CeO_2_	Calcination/ Ultrasonication	600	CeO_2_ fibers
IM-PtCe	[Pt(NH_3_)_4_](NO_3_)_2_-CeO_2_	Wet-impregnation	150	Pt-CeO_2_ fibers
ST-PtCe	K_2_PtCl_4_-CeO_2_	Solvothermal deposition	150	Pt-CeO_2_ fibers
IM-PtCeSi ^a^	IM-PtCeO_2_-SiO_2_	Sol-gel method	360	Core-sheath fibers (IM-Pt-CeO_2_ NF@SiO_2_)
ST-PtCeSi ^a^	ST-PtCeO_2_-SiO_2_	Sol-gel method	360	Core-sheath fibers (ST-Pt-CeO_2_ NF@SiO_2_)

^a^ Pt loading wt.% in core-sheath NFs is found to be 0.5% based on ICP-OES measurement.

**Table 2:** A perspective of different catalysts performance for RWGS based on Pt loading (%wt.). All reactions were performed in a fixed-bed reactor.

Catalyst	Structure	Pt Loading	Temperature	GHSV	$X_{{\mathrm{CO}}_{2}}$	$S_{\mathrm{CO}}$	Ref.
		%wt	°C	mL gcat^−1^ h^−1^	%	%	
Pt-CeO_2_ NF@SiO_2_	Core-sheath NFs	0.5	350	66,000	9	≈99	This work
Pt-Al_2_O_3_	Nanoparticles	0.0125–0.25	300	80,000	9	>99	[23]
Pt-TiO_2_	Nanoparticles	0.025 and 2	250	80,000	<2	100	[85]
Pt-CeO_2_	Nanorods	0.3	350	72,000	8.5	>98	[37]
Pt-TiO_2_	Nanoparticles	0.5	400	6000	15	≈98	[24]
Pt-CeO_2_	Nanoparticles	1	300	30,000	6.7	NA	[36]
Pt/20%CeO_2_-TiO_2_	Nanoparticles	1	300	12,000	6.5	NA	[86]
TiO_2_-supported Pt	Nanoparticles	1	300	12,000	20	NA	[87]
Pt-CeO_2_	NA	2	290	600,000	20	NA	[88]

## Data Availability

Data are contained within the article or Supplementary Information.

## Supplementary Materials

The following supporting information can be downloaded at: https://www.mdpi.com/article/10.3390/nano13030485/s1. Figure S1: XRD Rietveld refinement of (a) CeO_2_-NF with the lattice parameter of 5.411(15) Å, (b) IM-PtCeSi with the lattice parameter of 5.410(30) Å and 3.922(85) Å for CeO_2_ and Pt, respectively, and Pt weight fraction of 2.5(0.2) wt.% and (c) ST-PtCeSi with the lattice parameter of 5.410(14) Å and 3.923(47) Å for CeO_2_ and Pt, respectively, and Pt weight fraction of 19.2(0.5) wt.%. The lattice parameter of CeO_2_ was kept intact after introducing Pt, meaning the reduction of CeO_2_ occurred only on the surface, not in bulk. Figure S2: FTIR spectra of CeSi. Figure S3: STEM and EDS elemental mapping of IM-PtCeSi with 10% loading of CeO_2_ with Pt in bright field (BF) mode and mapping of elements, red color is Pt, green is Si and Blue is Ce. Figure S4: STEM and EDS elemental mapping of ST-PtCeSi with 10% loading of CeO_2_ with Pt. Figure S5: STEM of ST-PtCeSi after optimization of Pt with the Pt particle size distribution in ST-PtCeSi histogram plot (the average particle size is 3.8 nm); Equations (S1)–(S3): The fractions of Ce^4+^ and Ce^3+^. Figure S6: XPS for O 1s for all samples. Figure S7: Ce^3+^ species are present in the samples of CeO_2_-Com, CeO_2_-NF, IM-PtCe, and ST-PtCe which all are without the SiO_2_ sheath. Table S1: Peak positions, Ce oxidation state, and integrated area of all samples showed in Figure 7, compared to the reference peak positions and corresponded oxidation states. Table S2: Pt peaks with corresponded oxidation states occurred in Figure 8. Figure S8: CO_2_-TPD profile of IM-PtCeSi and ST-PtCeSi core-sheath NF catalysts. Figure S9: CO selectivity and CH_4_ selectivity at different reaction temperatures for two tested catalysts. Figure S10: Equilibrium conversion of CO_2_ at different temperature with different H_2_:CO_2_ compositions. Figure S11: TEM images of tested catalysts (a) and (b) IM-PtCeSi, (c) and (d) ST-PtCeSi. References [59,60,61,62,63,64,66,70] are cited in the Supplementary Materials.
