# Supplementary material for: Core-Sheath Pt-CeO2/Mesoporous SiO2 Electrospun Nanofibers as Catalysts for the Reverse Water Gas Shift Reaction
Source: Nanomaterials (Basel). 2023 Jan 25;13(3):485. doi: 10.3390/nano13030485 (PMC9921642; doi:10.3390/nano13030485)
Supplement: Supplementary file 1 [file nanomaterials-13-00485-s001.zip › nanomaterials-2162006-supplementary.pdf]

Supplementary Materials

Core-Sheath Pt-CeO_2/Mesoporous SiO_2 Electrospun Nanofibers as Catalysts for the Reverse Water Gas Shift Reaction

Aidin Nejadsalim [1,†], Najmeh Bashiri [2,3,†], Hamid Reza Godini [4], Rafael L. Oliveira [5], Asma Tufail Shah [1,6], Maged F. Bekheet [1], Arne Thomas [2], Reinhard Schomaecker [3], Aleksander Gurlo [1] and Oliver Görke [1,*]

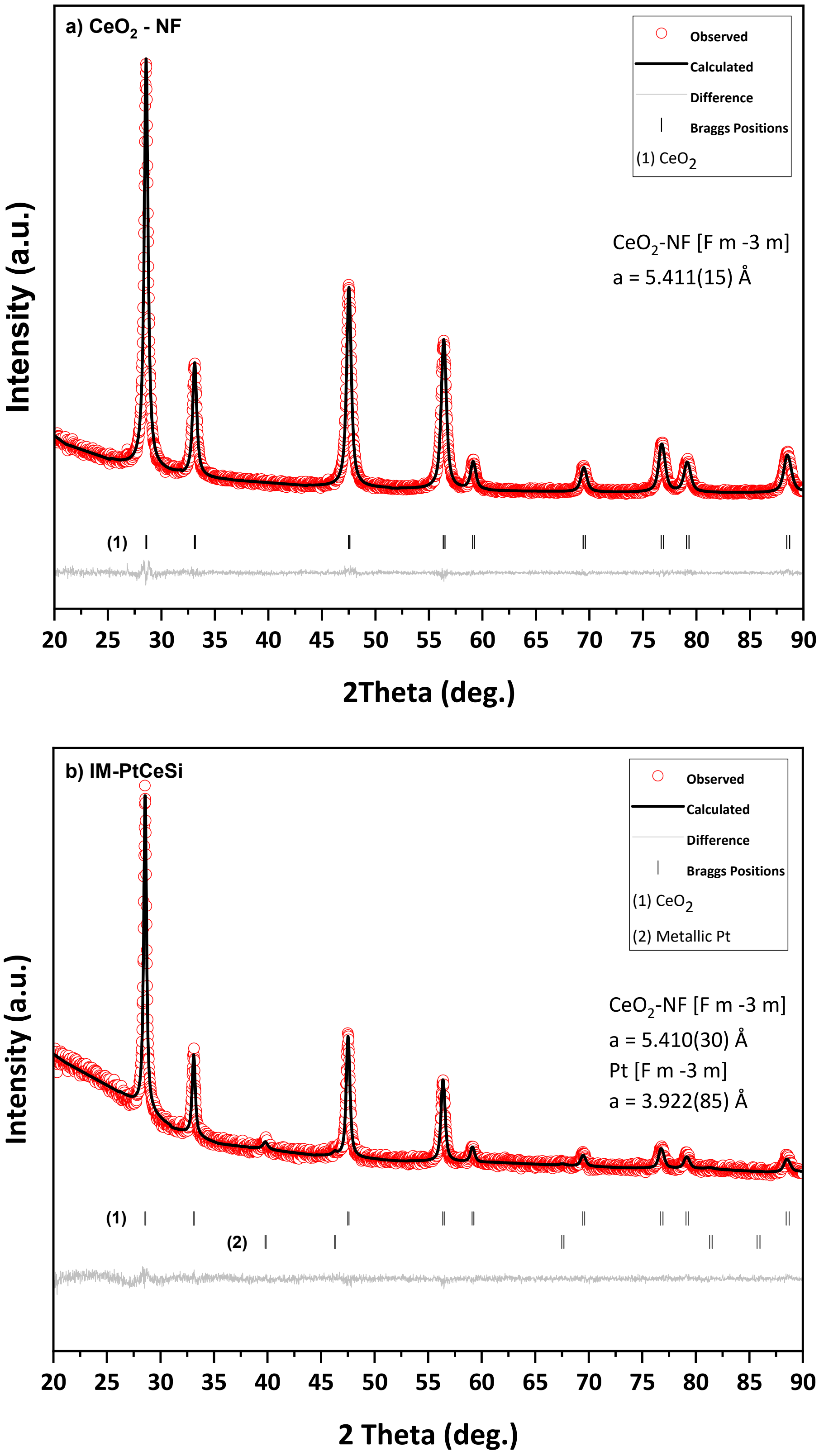

a) CeO2 - NF
Observed
Calculated
Difference
Braggs Positions
(1) CeO2
CeO2-NF [F m -3 m]
a = 5.411(15) Å
Intensity (a.u.)
(1)
20 25 30 35 40 45 50 55 60 65 70 75 80 85 90
2Theta (deg.)
b) IM-PtCeSi
Observed
Calculated
Difference
Braggs Positions
(1) CeO2
(2) Metallic Pt
CeO2-NF [F m -3 m]
a = 5.410(30) Å
Pt [F m -3 m]
a = 3.922(85) Å
Intensity (a.u.)
(1)
(2)
20 25 30 35 40 45 50 55 60 65 70 75 80 85 90
2 Theta (deg.)

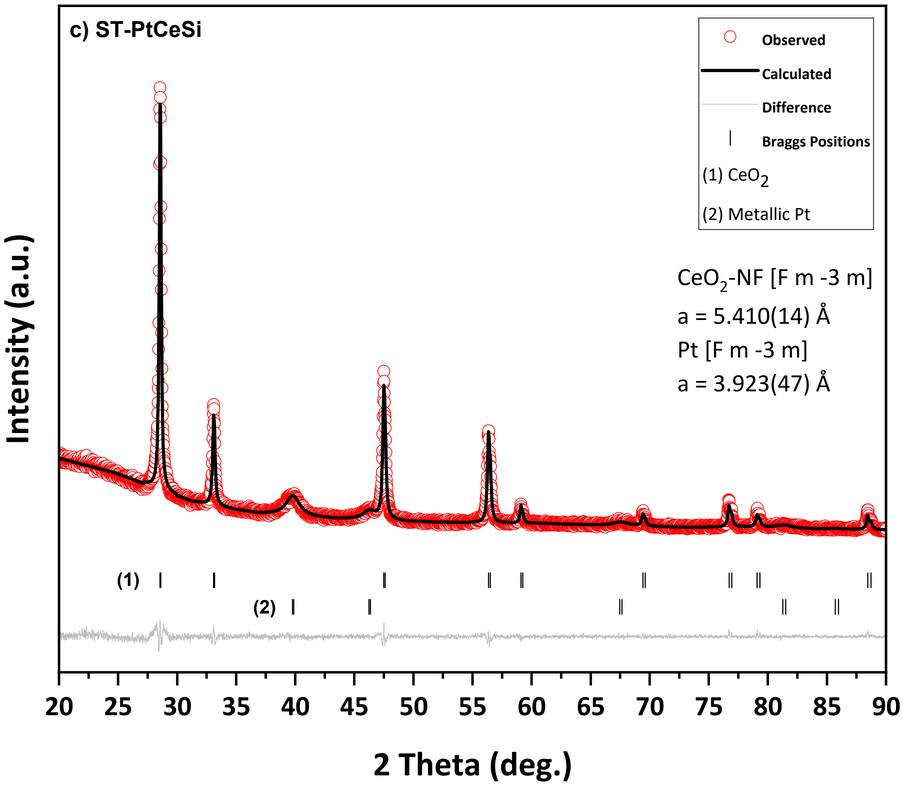

Figure S1. XRD Rietveld Refinement of (a) CeO_2-NF with the lattice parameter of 5.411(15) Å, (b) IM-PtCeSi with the lattice parameter of 5.410(30) Å and 3.922(85) Å for CeO_2 and Pt, respectively, and Pt weight fraction of 2.5(0.2) wt% and (c) ST-PtCeSi with the lattice parameter of 5.410(14) Å and 3.923(47) Å for CeO_2 and Pt, respectively, and Pt weight fraction of 19.2(0.5) wt%. The lattice parameter of CeO_2 was kept intact after introducing Pt meaning the reduction of CeO_2 was occurred only on the surface not in the bulk.

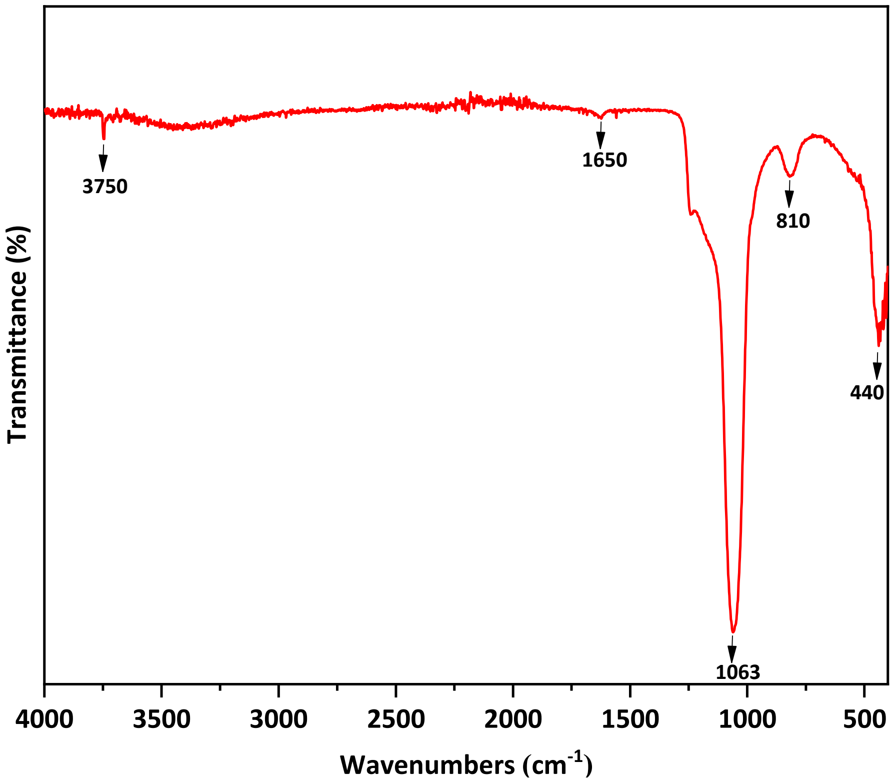

Figure S2. FTIR spectra of CeSi.

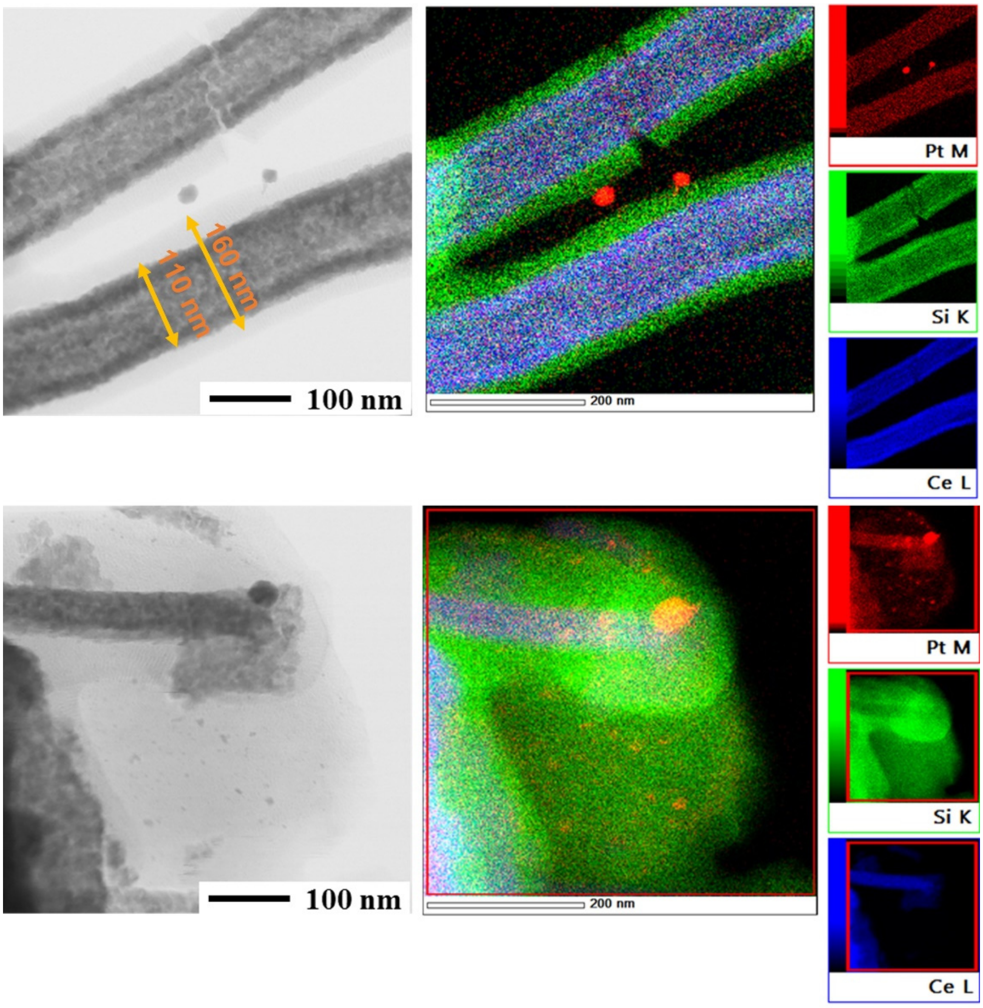

Figure S3. STEM and EDS elemental mapping of IM-PtCeSi with 10% loading of CeO_2 with Pt in bright field mode and EDS elemental mapping of elements, red color is Pt, green is Si and Blue is Ce.

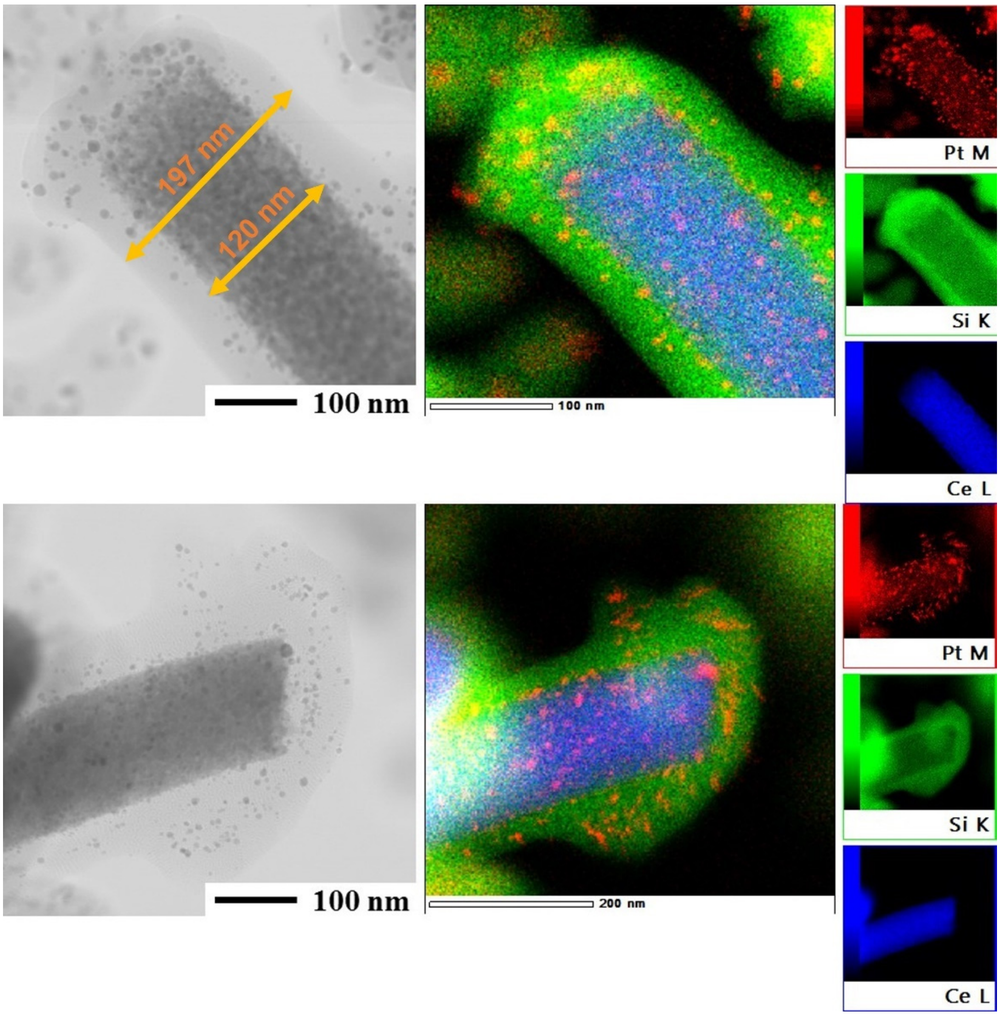

Figure S4. STEM and EDS elemental mapping of ST-PtCeSi with 10% loading of CeO_2 with Pt.

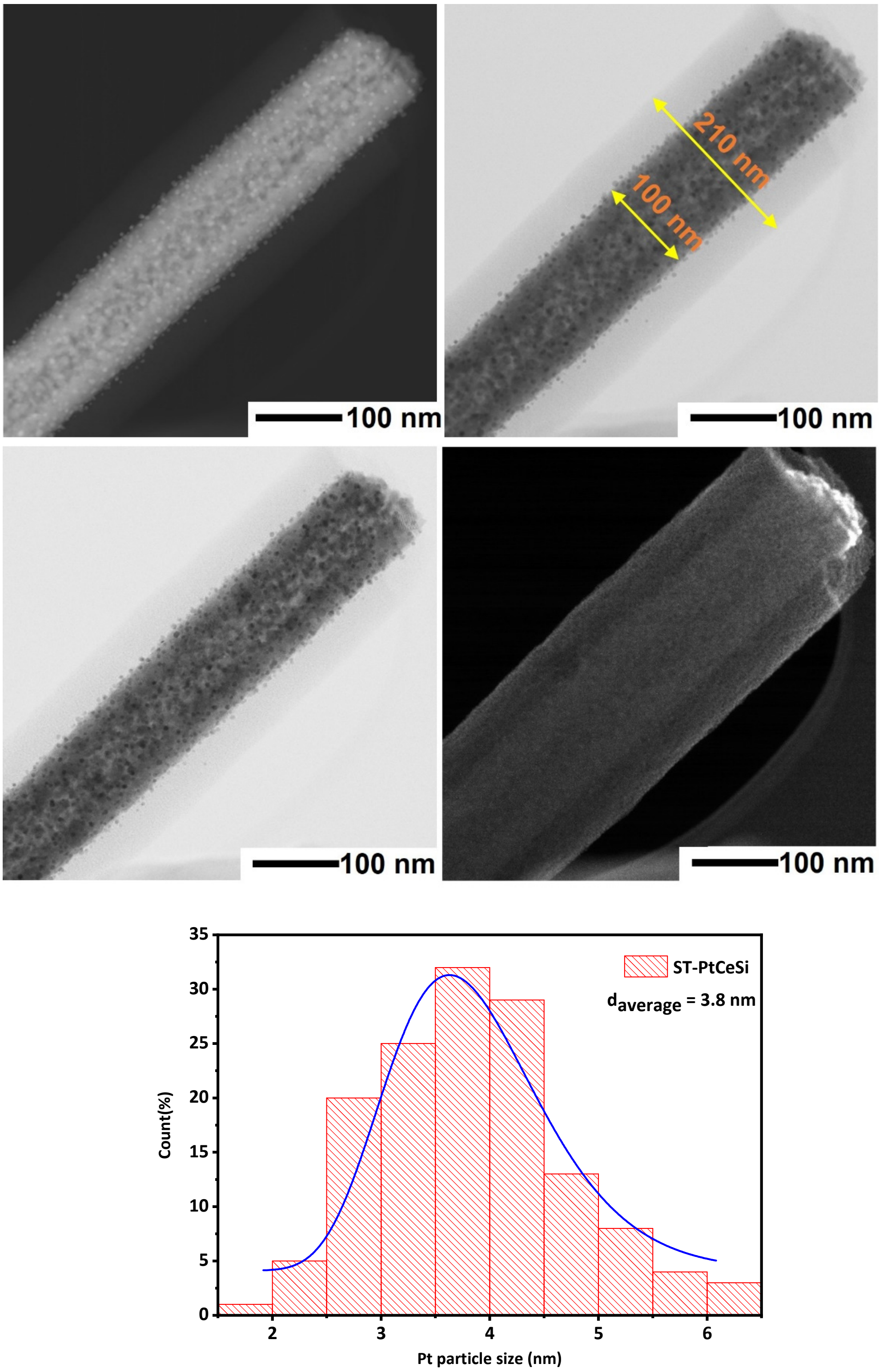

Figure S5. STEM of ST-PtCeSi after optimization of Pt with the Pt particle size distribution in ST-PtCeSi histogram plot (the average particle size is 3.8 nm).

The fractions of Ce^{4+} and Ce^{3+} were calculated using Equations (S1)-(S3) [66]:

$$V_{Ce^{4+}} = V(v) + V(u) + V(v'') + V(u'') + V(v''') + V(u''') \qquad (S1)$$

$$V_{Ce^{3+}} = V(u_0) + V(v_0) + V(v') + V(u') \qquad (S2)$$

$$\%Ce^{3+} = \frac{V_{Ce^{3+}}}{V_{Ce^{3+}} + V_{Ce^{4+}}} \times 100\,\% \qquad (S3)$$

Where V is the integrated peak area of the corresponding peaks.

XPS spectra related to O 1s are illustrated in Figure S6. Three characteristic peaks can be observed. The first feature appears at the peaks of 528.7-529.7 eV, which is attributed to oxygen bounded to Ce species of Ce^{3+} and Ce^{4+} lattice oxygen (O^{2-}, O_{α}). The other oxygen state occurs in the peak range of 531.1-531.8 eV that is assigned to the defect oxygen sites, oxygen vacancy (O^{-}, O_{β}) [59,60]. The third peak at 532.1-532.8 eV is accredited to the oxygen anions (O_2^{-}) seated near oxygen vacancy [61-63] which might be OH groups and adsorbed water [64]. The lattice oxygen peak appeared for all samples without SiO_2 sheath layer.

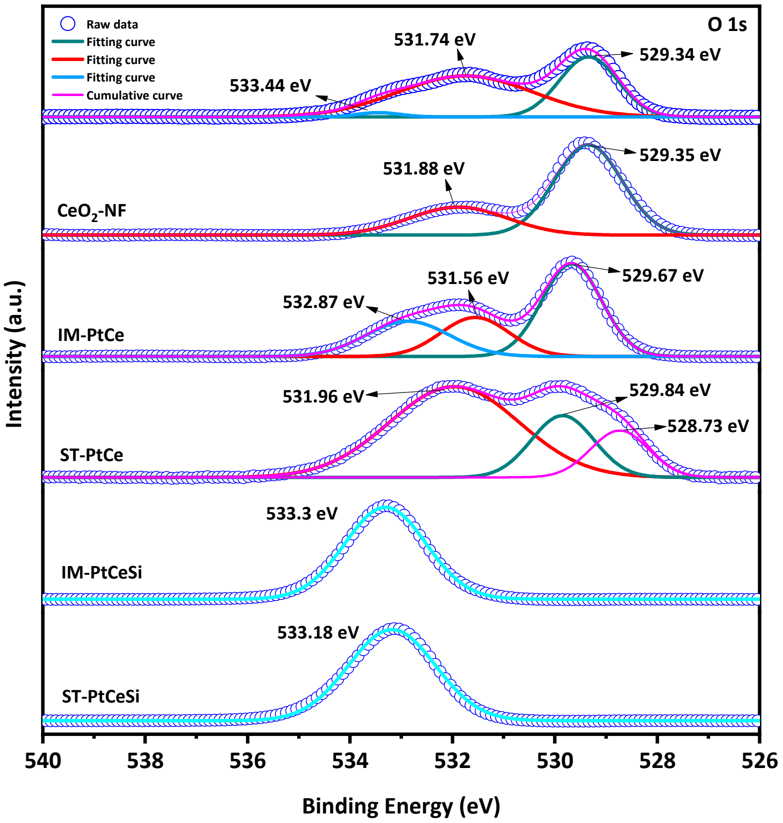

Figure S6. XPS for O 1s for all samples.

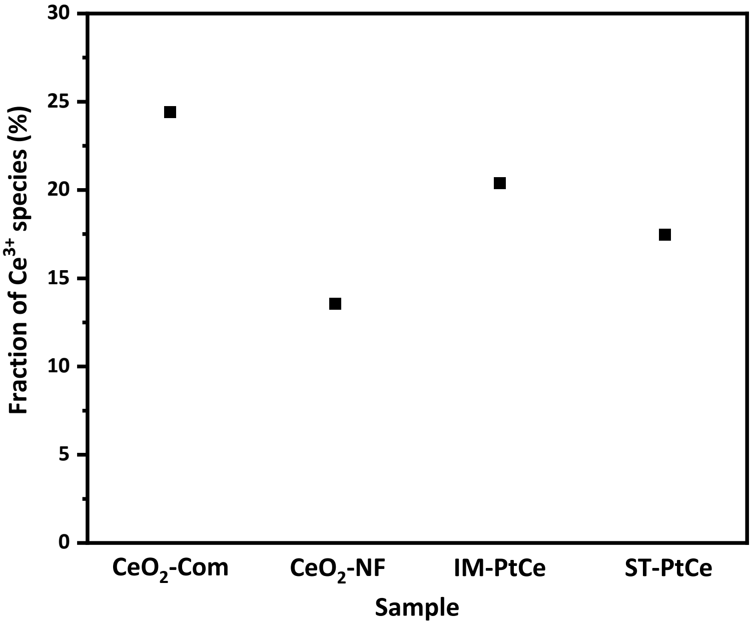

Figure S7. Ce^{3+} species are present in the samples of CeO_2-Com, CeO_2-NF, IM-PtCe, and ST-PtCe which all are without the SiO_2 sheath.

Table S1. Peak positions, Ce oxidation state, and integrated area of all samples showed in Figure 7, compared to the reference peak positions and corresponded oxidation states from [70].

Ce oxidation state	Spin-orbit splitting	Peak assignment	Samples													
			Reference	CeO_2-Com		CeO_2-NF		IM-PtCe		ST-PtCe		IM-PtCeSi		ST-PtCeSi	
			BE (eV)	BE (eV)	Peak area	BE (eV)	Peak area	BE (eV)	Peak area	BE (eV)	Peak area	BE (eV)	Peak area	BE (eV)	Peak area
**Ce^{4+}**	Ce $3d_{3/2}$	u′′′	916.7	916,8	45122.4	916,64	70186.5	916,9	77433.0	916,58	24232.7	916.9	7946.5	917.4	21922.7
	Ce $3d_{3/2}$	u′′	907.45	907,5	37331.7	907,38	56452.0	907,71	70075.7	907,1	29523.0	-	-	-	-
	Ce $3d_{3/2}$	u	901.06	900,7	27654.2	900,85	55549.7	901,2	64024.0	900,87	25965.3	-	-	-	-
	Ce $3d_{5/2}$	v′′′	898.4	898,1	65783.2	898,24	116168.0	898,5	114887.1	898,18	38272.7	-	-	-	-
	Ce $3d_{5/2}$	v′′	888.85	888,87	79816.0	888,98	121170.0	889,31	122069.8	888,7	42533.0	-	-	-	-
	Ce $3d_{5/2}$	v	882.6	882,3	88332.5	882,45	150918.0	882,8	129545.3	882,47	51702.1	-	-	-	-
**Ce^{3+}**	Ce $3d_{3/2}$	u′	904.05	903,1	54816.8	902,95	56911.8	903,2	73984.0	903,17	13148.0	904.6	36792.0	905.2	97679.8
	Ce $3d_{3/2}$	u_0	898.9	-	-	-	-	-	-	-	-	900.3	17670	900.7	49955.7
	Ce $3d_{5/2}$	v′	885.45	884,7	29704.3	884,55	43956.3	884,8	58227.5	884,77	14328.4	886.2	50286.6	886.7	134797.8
	Ce $3d_{5/2}$	v_0	880.6	-	-	-	-	-	-	-	-	882.0	28526.8	882.5	73278.7

Table S2. Pt peaks with corresponded oxidation states occurred in Figure 8.

Pt oxidation state	Spin-orbit splitting	Samples			
		IM-PtCe	IM-PtCeSi	ST-PtCe	ST-PtCeSi
		BE (eV)	BE (eV)	BE (eV)	BE (eV)
**Pt^{2+}**	Pt $4f_{7/2}$	72.8	72.6	73.38	-
	Pt $4f_{5/2}$	76.15	75.95	76.73	-
**Pt^{0}**	Pt $4f_{7/2}$	-	-	70.96	71.25
	Pt $4f_{5/2}$	-	-	74.31	74.6

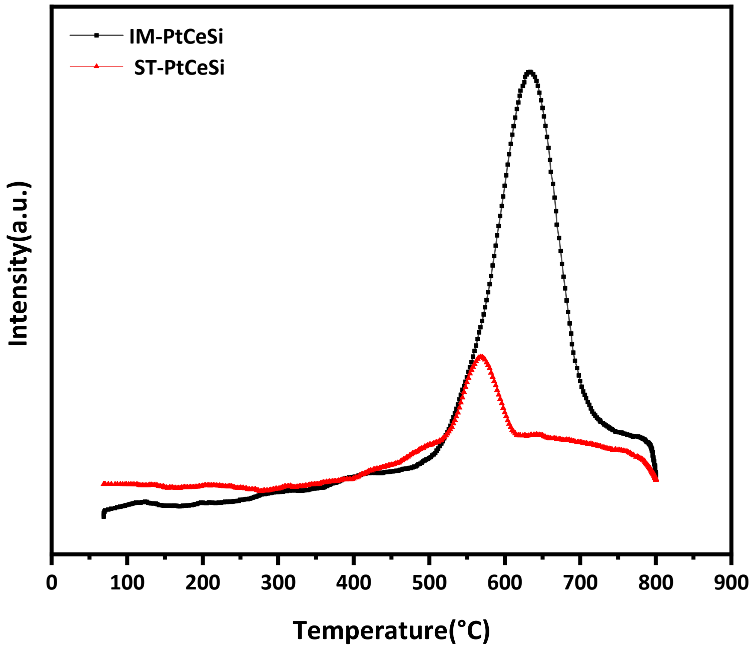

Figure S8. CO_2-TPD profile of IM-PtCeSi and ST-PtCeSi core-sheath NF catalysts.

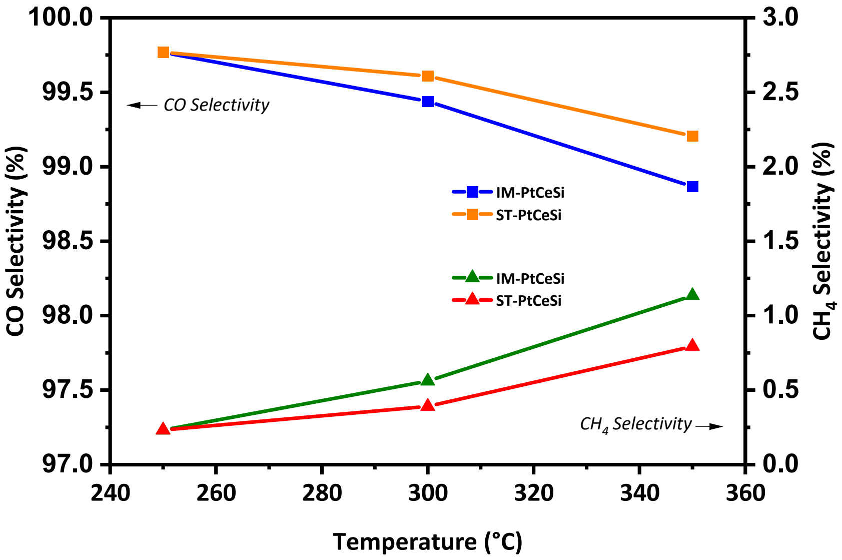

Figure S9. CO selectivity and CH_4 selectivity at different reaction temperatures for two tested catalysts.

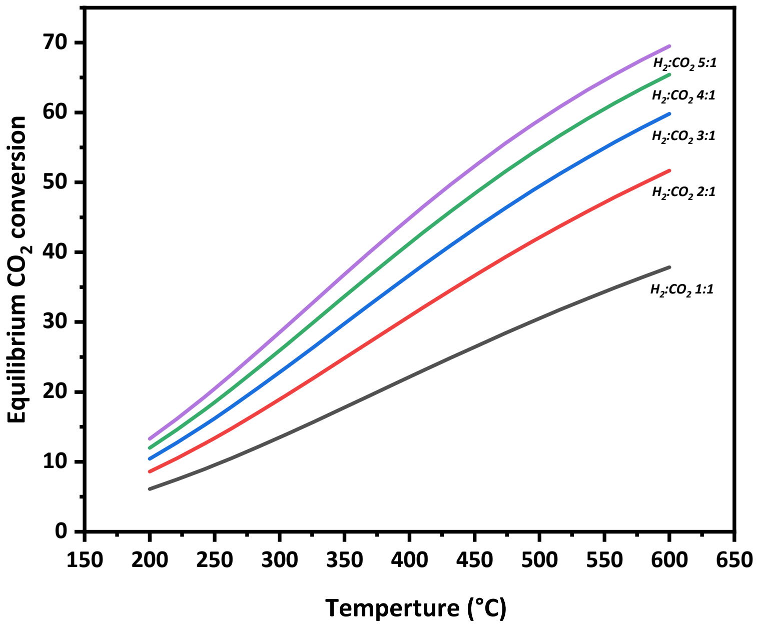

Figure S10. Equilibrium conversion of CO_2 at different temperature with different $H_2:CO_2$ compositions.

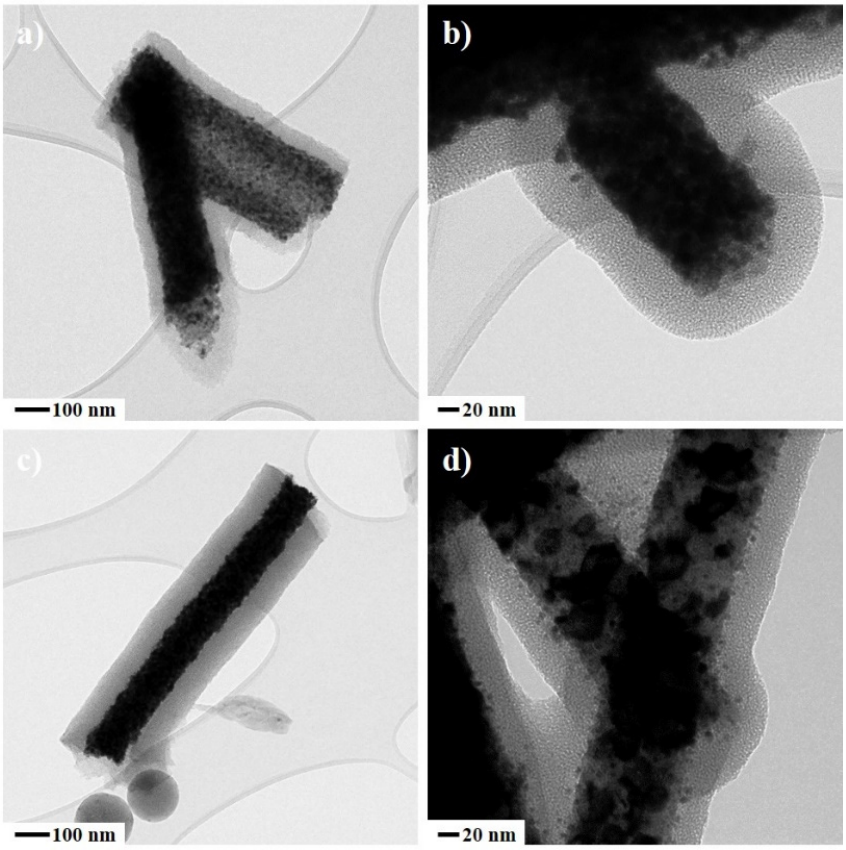

Figure S11. TEM images of tested catalysts (a) and (b) IM-PtCeSi, (c) and (d) ST-PtCeSi.
